# T2-Fluid-Attenuated Inversion Recovery (FLAIR) Mismatch as a Novel Specific MRI Marker for Adult Low-Grade Glioma (LGG): A Case Report

**DOI:** 10.7759/cureus.29457

**Published:** 2022-09-22

**Authors:** Rahaf M Slaghour, Rema A Almarshedi, Arwa M Alzahrani, Fahad Albadr

**Affiliations:** 1 Medicine, King Abdulaziz University Faculty of Medicine, Jeddah, SAU; 2 Medicine, University of Hail College of Medicine, Hail, SAU; 3 Medicine, King Saud Medical City, King Saud University, Riyadh, SAU; 4 Radiology and Medical Imaging/Neuroradiology, King Saud Medical City, King Saud University, Riyadh, SAU

**Keywords:** isocitrate dehydrogenase-mutated, grade 2 glioma, astrocytoma, low grade gliomas, t2-flair mismatch

## Abstract

Astrocytic tumors are primary central nervous system tumors. They are the most common tumors arising from glial cells. In the new WHO classification 2021, adult-type diffuse astrocytic gliomas subdivide into isocitrate dehydrogenase (IDH)-mutant astrocytoma, IDH-mutant and 1p/19q-codeleted oligodendroglioma, and IDH-wildtype glioblastoma. The T2-fluid-attenuated inversion recovery (FLAIR) mismatch sign describes the MRI appearance of IDH-mutant astrocytoma, it is considered a highly specific radiogenomic signature for diffuse astrocytoma, as opposed to other lower-grade. MRI is the first and most accurate diagnostic tool for low-grade gliomas (LGGs). It is particularly helpful in distinguishing a diffuse astrocytoma from an oligodendroglioma that will not demonstrate T2-FLAIR mismatch. The tumor displays a hyperintense signal on T2-weighted images and a hypointense signal on T2-weighted FLAIR images, which distinguishes it from other types of diffuse gliomas. We report a case of a 29-year-old female patient who was diagnosed with IDH-mutant 1p/19q-non-codeleted diffuse astrocytoma based on MRI T-2 FLAIR mismatch sign, which is confirmed by the molecular analysis in the pathology lab. Our aim of this report is to confirm the power of the MRI findings in the diagnosis of glioma genotypes and to assess neurosurgeons in the preoperative surgical planning.

## Introduction

Low-grade gliomas (LGGs) are tumors arising from the supporting glial cells of the central nervous system, accounting for 15% of all primary brain tumors. These brain tumors have been graded within type from Grades 2 to 4 according to the 2021 WHO classification [1-3].

Isocitrate dehydrogenase (IDH)-mutant diffuse astrocytoma is a subtype of LGG that primarily affects young adults with a mean age of 39.4 years and standard deviation of 17.3. Patients with this condition usually present with seizures and worsening headaches. The temporal and frontal lobes are predominantly involved, and the tumor resection significantly improves overall survival. Patients with IDH-mutant diffuse astrocytoma have a 3.9 to 10.8 years of median survival time [3].

The “T2-fluid attenuated inversion recovery (FLAIR) mismatch” sign can identify a lesion, which appears on T2- weighted images as a high signal and on T2- weighted FLAIR sequences a low signal, except for a high signal in the peripheral rim [4]. It is a promising radiognomic marker; it is detectable during clinical practice on routine MRI that allows for pre-operative diagnosis of IDH-mutant astrocytomas with a high level of specificity [5].

Diffuse LGGs are diagnosed based on the histopathological and molecular features, such as IDH 1 and IDH 2 gene mutations and the codeletion of short and long arms of chromosomes 1 and 19, as per the WHO 2021 classification [2].

The treatment of IDH-mutant diffuse astrocytoma is highly dependent on the T2-FLAIR mismatch sign, and a 2021 meta-analysis showed a pooled specificity of 100% for tumors with an IDH-mutant, 1p and 19q non-codeleted variant, which makes it reliable in the diagnosis of IDH-mutant diffuse astrocytoma and in distinguishing it from other diffuse gliomas, although the pooled sensitivity was 42% [6].

Here, we present a case study of a patient with IDH-mutant 1p and 19q-non-codeleted diffuse astrocytoma that was diagnosed pre-operatively with the help of MRI.

## Case presentation

A 29-year-old Saudi Arabian woman, newly diagnosed with an undifferentiated connective tissue disease based on positive antinuclear antibodies, anti-Ro/SSA, and anti-La/SSB. However, she had negative double stranded DNA and rheumatoid factor. She was on hydroxychloroquine 200 mg and regularly follows up with the rheumatology clinic.

She was admitted for involuntary jerky movements on her left side of body while sleeping, in addition to headache, numbness and blurred vision for one year. Upon examination, her cranial nerves were intact, power was five out of five on all limbs, sensations were intact, and gait was normal.

**Figure 1:**
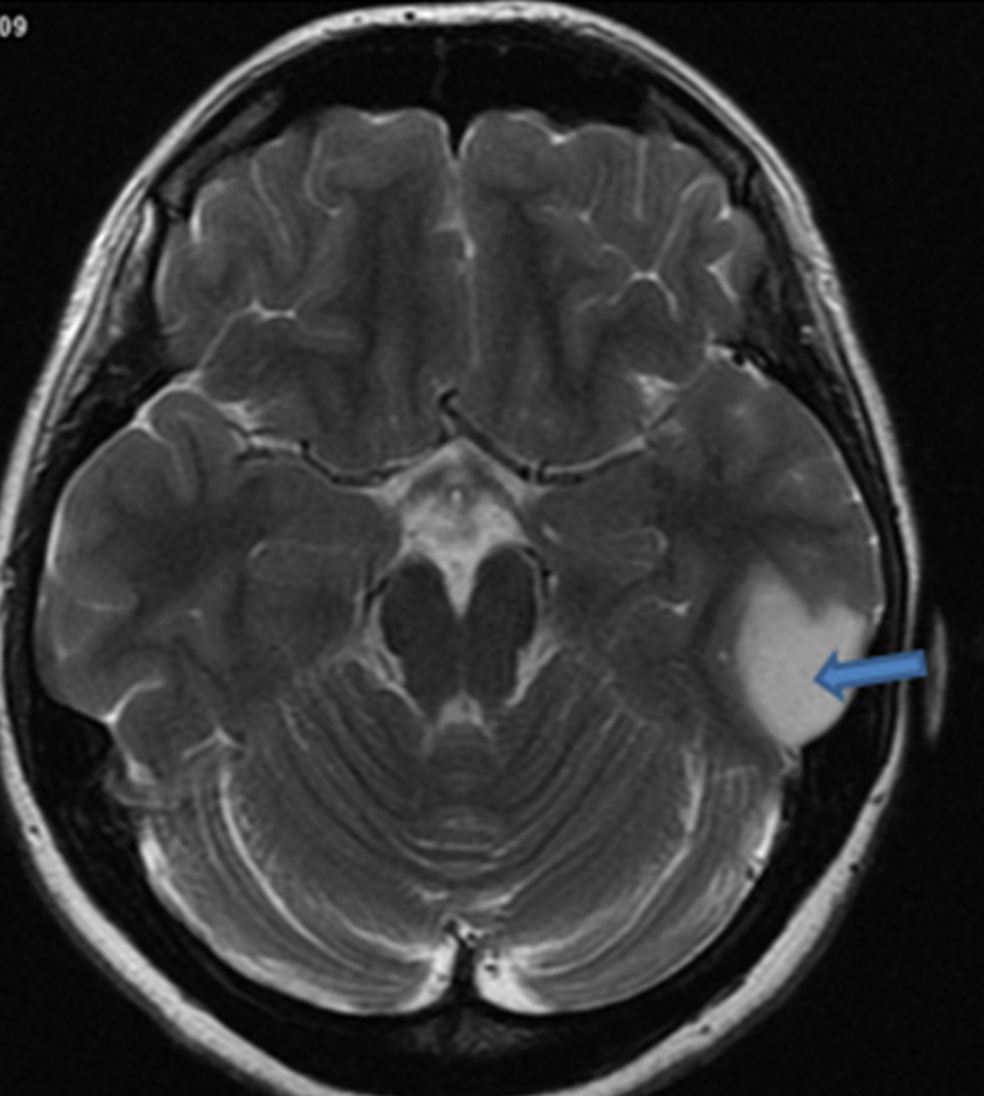
Axial T2 weighted image Left temporal cortical and subcortical lesion with a homogenous hyperintense signal on T2 weighted imaging on the central aspect of the lesion. No peritumoral edema or mass effect.

The non-contrast MRI studies revealed a left temporal cortical and subcortical lesion with a homogenous hyperintense signal on T2 weighted imaging in the central aspect of the lesion. No peritumoral edema or mass effect was observed (Figure 1).

**Figure 2:**
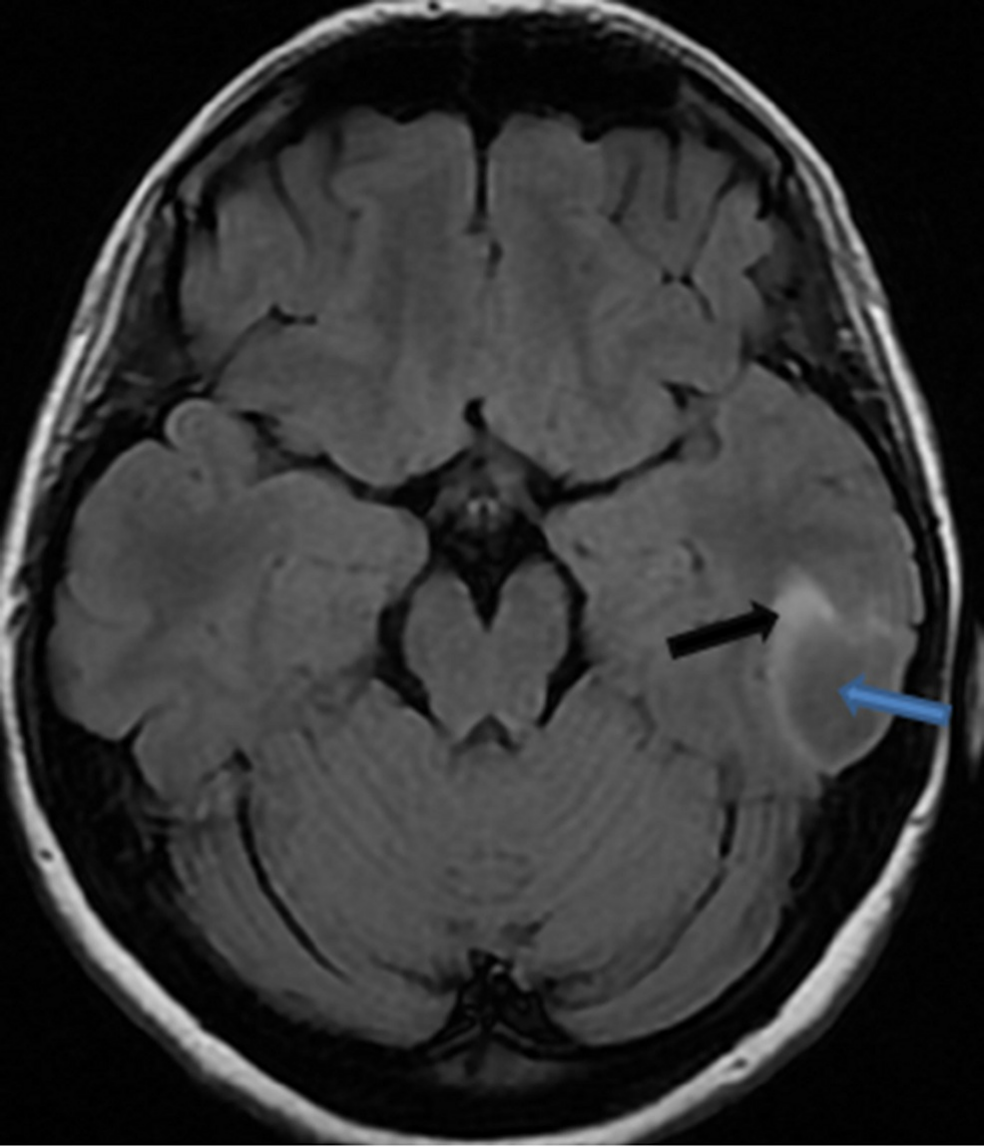
Axial FLAIR Left temporal cortical and subcortical lesion had incomplete suppression of the central aspect of the lesion (blue arrow) and a high-signal peripheral rim (black arrow).

On FLAIR images, the left temporal cortical and subcortical lesions had central suppression of the central aspect of the lesion and a high-signal peripheral rim (Figure 2).

**Figure 3:**
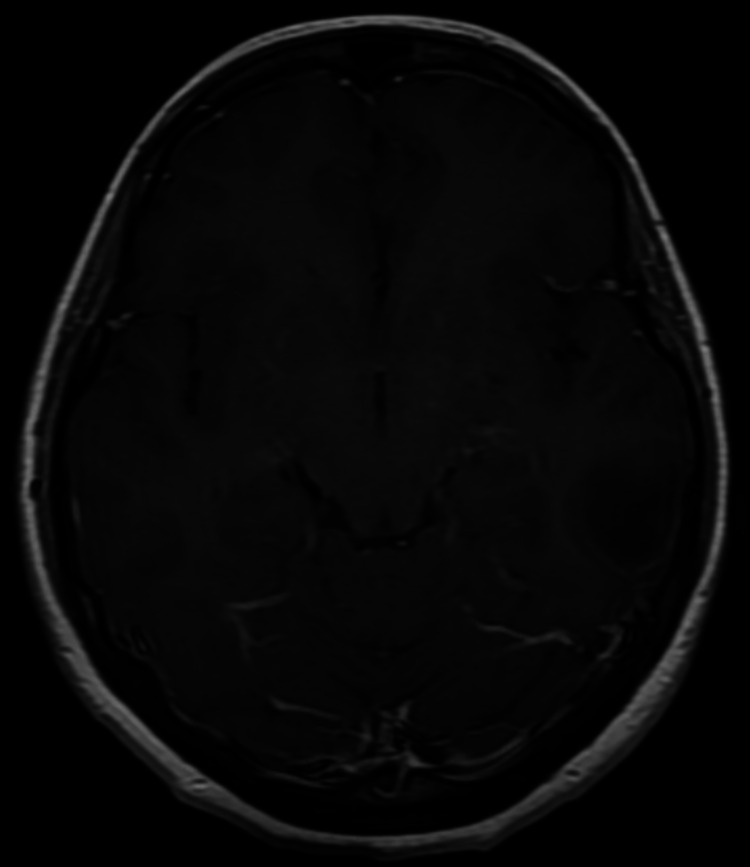
Axial post contrast T1 weighted image The lesion was well-defined, with no internal or peripheral enhancement. No reaction to the adjacent bone or meningeal enhancement was observed.

The lesion was well-defined, with no internal or peripheral enhancement. No reaction to the adjacent bone or meningeal enhancement was observed (Figure 3).

Two weeks prior to surgery hydroxychloroquine was stopped and she underwent left temporal craniotomy and excision of the low-grade lesion, and a sample was taken for histopathology and molecular analysis.

The sample confirmed the cellular infiltration of the LGG. It exhibited round nuclear contours and distinct cellular borders based against the background of scattered capillary-sized vessels, similar to ‘chicken wire appearance’. The tumor cells were positive for glial fibrillary acidic protein (GFAP) and synaptophysin and exhibited a focal to patchy reactivity pattern suggestive of p53 among scattered neurons. The Ki-67 proliferative index was low (focally, up to 4%). These features are consistent with those of WHO grade 2 gliomas. Genetic analysis was positive for the IDH 1 R132H gene mutation and negative for the 1p and 19q codeletion.

Postoperatively, the patient was admitted to an intensive care unit for observation, which was uneventful. Day one post-operative MRI showed early postsurgical changes after gross resection of the left temporal mass, and no residual tumor was identified. She was shifted to the ward and kept under neuro-observation, where in the course of her stay the jerky involuntary movements stopped, her headache improved, and daily neurological examination showed intact cranial nerves, coherent speech, no focal neurological lesion signs, she had good power of limbs bilaterally and her pupils were equal and reactive to light. The patient was discharged home and continued hydroxychloroquine.

**Figure 4:**
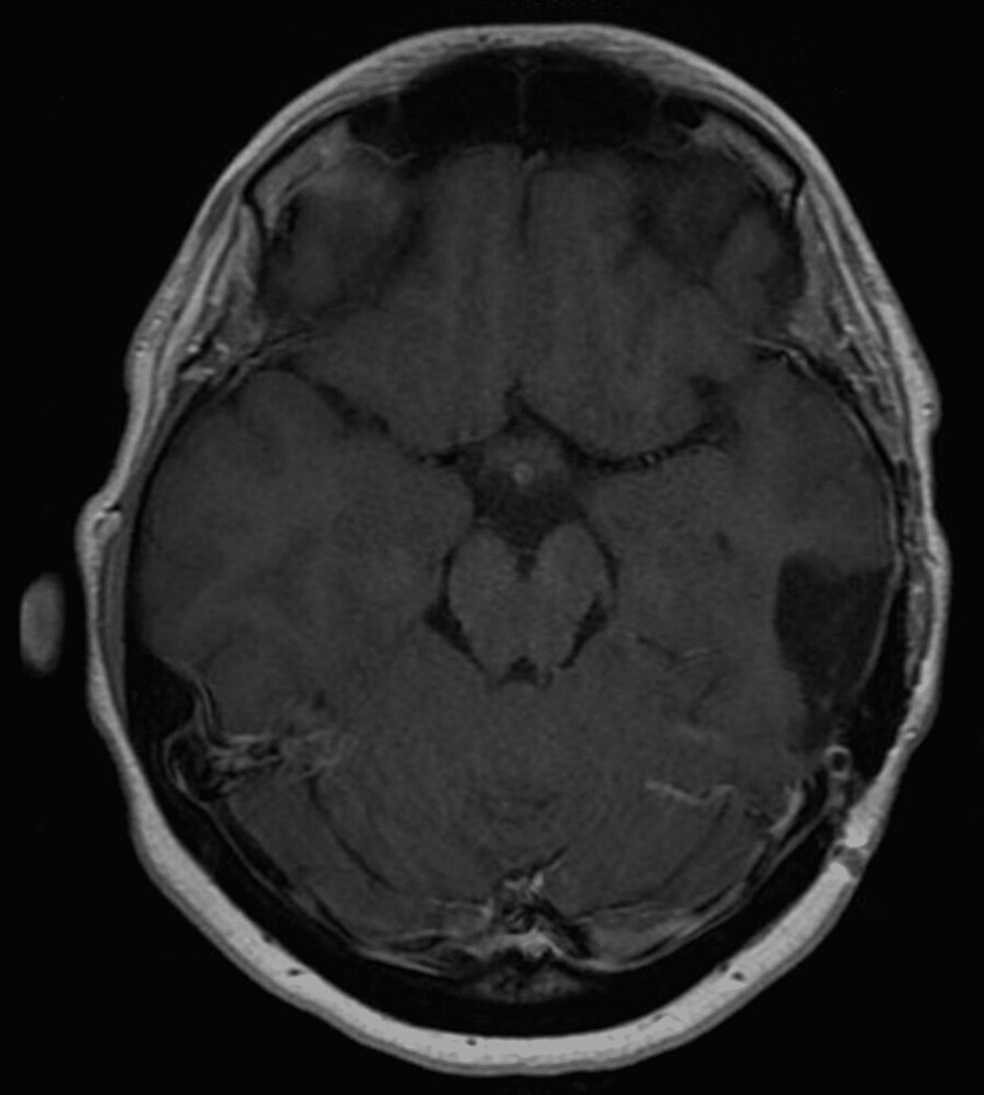
Follow-up axial post contrast T1 There is complete resection of the mass without evidence of recurrent or residual tumor.

She received one month of daily 100 mg of temozolomide (TMZ) and radiotherapy of 5,400 centigray (cGy) in 30 fractions to the left temporal lobe by the oncologists. During her 10th month follow up, MRI showed stable surgical changes in the left temporal lobe with no evidence of tumor recurrence (Figure 4).

## Discussion

The diagnosis of LGGs is made using imaging, histopathology, and molecular diagnostic methods. The current diagnostic imaging modality of choice is MRI, which indicates a homogeneous tumor with hypointense signal on T1-weighted sequences and hyperintense signal on T2-weighted and FLAIR sequences [7]. Across the literature the T2 FLAIR mismatch has been found to be highly specific in IDH mutant non-codeleted astrocytoma but low sensitivity. A meta-analysis reported the mismatch sign 100% specific for identifying tumors with an IDH-mutant, 1p and 19q non-codeleted status. However, it has a low sensitivity of 42% [6]. Adamou et al study, showed the diagnostic accuracy for IDH mutant non-codeleted LGGs with specificity and sensitivity of 97% and 40%, respectively [8].

The clinical implications of T2 mismatch sign in the setting of LGGs have been limited. The mismatch sign under strict criteria is able to diagnose IDH mutant non-codeleted astrocytoma preoperatively in adults with suspicious intracranial masses suggestive of diffuse gliomas. In addition, the T2 mismatch sign can be used as a predictive tool for the IDH status regardless of the 1p/19q status, where IDH wild type LGGs can be excluded with high confidence [4]. In this case, the MRI findings were used preoperatively as a noninvasive tool to diagnose the patient LGG and later confirmed by the histological and genetic analysis.

Various studies differ in the significance of the mismatch alongside other parameters. Kapsalaki et al. reported that implementing additional co-registration imaging parameters have no significance in improving the diagnostic accuracy of the mismatch sign [9]. Another study found that mismatch sign has no correlation with symptoms, preoperative, and postoperative volume, type, and extent of surgery or survival [10]. However, Juratli et al. observed that T2 FLAIR mismatch sign in patients younger than 40 years old and a tumor size larger than 6 cm are strong factors related to IDH mutant astrocytoma with respective specificity and positive predictive value of 96% and 88% [11].

The IDH 1 mutation in LGG has recently been identified as a common early mutation, which is rarely observed in primary glioblastoma. Several studies have reported the role of IDH 1 mutations in the malignant progression of LGG. This indicates that a diffuse glioma has less aggressive behavior, favorable symptoms, and is less likely to undergo malignant transformation [12].

Generally, the main treatment options of LGGs are surgical resection, followed by radiation and chemotherapy [13]. Huang et al. conducted a study on 230 IDH mutant patients regarding efficacy of chemotherapy, radiotherapy, or chemoradiotherapy on tumor growth, of which 95 underwent scan pre- and post-treatment. Across these patients, the growth rate before treatment and post treatment, respectively, were 26.63% and -15.24%. 53 patients were treated with TMZ monotherapy and the pre-treatment growth rate was 24.48% and was reduced to -14.87% post treatment. Additionally, the 95 patients pre-treatment and post-treatment images were also grouped based on 1p/19q status as codeleted and non-codeleted, which showed significant decline in growth rate [14]. Furthermore, a systematic meta-analysis reported the impact of survival during the management course of patients with LGG. Total resection correlated with a higher survival rate, as well as adjuvant radiotherapy combined with chemotherapy was associated with progression free survival (PFS) benefits at five and 10 years. Chemotherapy showed a higher benefit for IDH 1 mutant glioma than for wild type glioma. Fractionated radiotherapy demonstrated PFS benefits at two, five, and 10 years [15].

## Conclusions

The T2 flair mismatch is a highly specific non-invasive radiological marker for diagnosing IDH-mutant non-codeleted astrocytoma in adult patients, particularly younger than 40 years old. It also able to predict the IDH status and exclude IDH wild. This non-invasive MRI finding can aid in the treatment decision processes in a significant proportion of patients presenting with non-enhancing, LGG-like lesions.
